# Targeting a specific subset of neutrophils to mitigate cardiac reperfusion injury

**DOI:** 10.21203/rs.3.rs-8273505/v1

**Published:** 2025-12-12

**Authors:** Abhalaxmi Singh, Andrew Stuart, Sreeparna Chakraborty, Asrar Malik, Kurt Bachmaier

**Affiliations:** University of Illinois, Chicago; University of Illinois, Chicago; University of Illinois, Chicago; Cell Biologics; University of Illinois, Chicago

**Keywords:** sterile inflammation, percutaneous coronary interventions, neutrophil heterogeneity, acute myocardial infarction, coronary patency, albumin nanoparticles, piceatannol

## Abstract

Ischemia reperfusion (IR)-induced oxidative stress and inflammation contribute to morbidity and mortality of acute coronary syndrome. Ischemia results in profound hypoxia and tissue dysfunction and subsequent reperfusion further aggravates ischemic cardiac tissue damage. In cardiac IR injury, neutrophils are involved both in causing cardiomyocyte death and in preserving heart tissue homeostasis. We tested the hypothesis that neutrophil subpopulations show distinct functions in the pathogenesis of cardiac IR injury and that their functional heterogeneity can be exploited in subset-specific pharmacological intervention to prevent IR-induced myocardial tissue damage and functional deterioration. Cardiac IR-injury in a mouse model was characterized by the presence of two distinct heart-inflammatory subsets of neutrophils, one that specifically endocytosed albumin nanoparticles (ANP^high^) and one that endocytose few or no ANP (ANP^low^). The two subsets had very distinct inflammatory phenotypes. ANP^high^ neutrophils expressed significantly greater amounts of inflammatory mediators, such as Il-1b and Ccl3, than ANP^low^ neutrophils. Targeting the Spleen tyrosine kinase (Syk) specifically in ANP^high^ neutrophils post IR reduced cardiac neutrophilic and mononuclear inflammation and drastically decreased infarct size, and prevented the deterioration of cardiac function. Targeting the Syk pathway specifically in a defined subset of neutrophils is a feasible therapy for cardiac IR injury.

Standard treatment for acute myocardial infarction (MI) is timely restoration of blood flow to ischemic tissues using percutaneous coronary intervention and thrombolytic therapies (1–5). Reperfusion of ischemic tissues can exacerbate damage by inducing inflammation, reactive oxygen species production, and microvascular dysfunction (6).

Neutrophils contribute to lethal injury of coronary vascular endothelial cells and cardiomyocytes after reperfusion (7). IR-injury causes emergency myelopoiesis (8, 9). Neutrophil-derived alarmins (S100A8 and S100A9), and danger-associated molecular patterns (DAMPs), signaling through Toll-like receptor 4 and the Nod-like receptor family pyrin domain-containing 3 inflammasome (NLRP3), serve to prime and promote interleukin-1 (IL-1) β secretion (9, 10). IL-1β/MyD88 signaling induces granulocyte colony stimulating factor (G-CSF) upregulation and consequently the expansion of pluripotent hematopoietic stem cells (11). The ischemic myocardium releases arachidonate and complement-derived chemotactic factors such as leukotriene B4 and C5a, which recruit and activate neutrophils, monocytes and macrophages (12, 13). Activated neutrophils induce NADPH oxidase complex-derived reactive oxygen species (ROS), such as O_2_·^−^ and H_2_O_2_, which further induce formation of OH· and hypochlorous acid (13–16). In patients, targeted PET imaging of chemokine receptor CXCR4 expression demonstrated CXCR4-expressing cell infiltration after cardiac reperfusion (17) suggestive of neutrophil sequestration. In patients with acute coronary syndrome, higher neutrophil counts seen on admission and after revascularization correlated with major adverse cardiovascular disease outcomes (10). Firmly adherent neutrophils, releasing ROS and proteolytic enzymes (16, 18), damage the coronary vascular endothelium and promote the thromboembolization of microvessels, generating no-reflow areas and secondary ischemia (19).

Therefore, removing neutrophils from the systemic or coronary circulation or the prevention of adhesion molecule-dependent interactions with the endothelium to mitigate neutrophil sequestration to the infarcted areas has been attempted experimentally and clinically. However, clinical studies of targeting Mac-1 expressing cells (with recombinant humanized monoclonal antibody to CD18 (Mac1 or LFA-1) or injections of antibodies to all isoforms of CD18) have shown negative results (20–22) and anti-neutrophilic therapeutic strategies have been largely abandoned in the clinic (23). Moreover, experimental depletion of neutrophils before injury-induction impaired cardiac recover (24) suggesting that neutrophils in addition to causing in jury are required for proper myocardial recovery after IR. The rationale for these studies was that cardiac inflammation induced by therapeutic reperfusion is predominantly driven by neutrophils (25–28) but, paradoxically, indiscriminate targeting of neutrophils is ineffective therapeutically (29). Targeting neutrophils specifically based of their distinct functional properties, rather than cell surface marker expression or (as yet unidentified) specific transcriptional programs (30, 31), seemed more feasible. For these reasons, we attempted to identify the specific neutrophil subset involved in cardiac IR injury based on the previously reported propensity of endothelial-adherent neutrophils to endocytose albumin nanoparticles (32–34).

## Results

### Albumin-based nanoparticles are differentially endocytosed by neutrophils in cardiac IR injury

**Figure 1:**
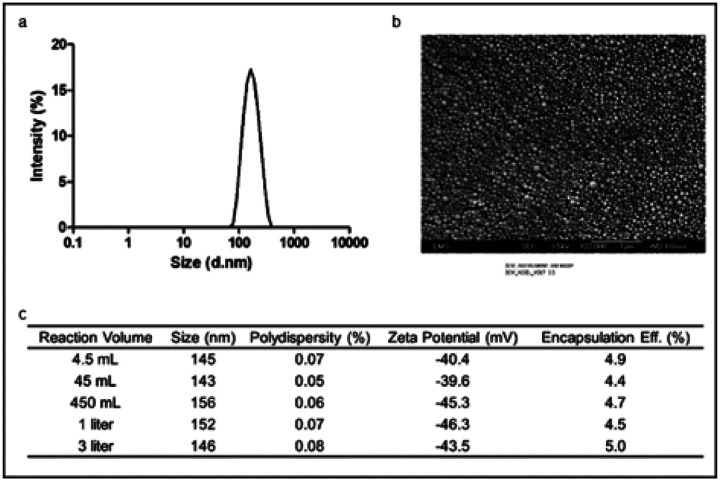
Human serum albumin (HSA)-based nanoparticles used for targeted drug delivery. Albumin nanoparticles (ANP) were synthesized using a solvent desolvation technique. ANP and piceatannol containing PANP size was characterized by light scatter **(a)** and scanning electron microscopy **(b)** and found to be 140–150nm in diameter with a polydispersity value of <0.1 and zeta potential < −40mV **(c)**.

In ischemia-reperfusion (IR) injury, both in experimental models and human patients, neutrophils are a key cellular component of the inflammatory response (35). We attempted to characterize neutrophils by their ability to endocytose albumin nanoparticles (33). For the synthesis of nanoparticles, we used human serum albumin (HSA), modifying a published method with bovine serum albumin (BSA) (32) (Fig. 1). BSA and HSA are highly homologous (36) but HSA-nanoparticles are more suitable for potential clinical applications (37). For use in flow cytometry, we synthesized nanoparticles loaded with Alexa Fluor^®^ 647 succinimidyl ester (AF647 (50μg) that showed bright and stable fluorescence (Extended data Fig. 1). After the desolvation reaction, the HSA-nanoparticle solutions were washed, and re-suspended in deionized water. These reactions yielded nanoparticles with a hydrodynamic diameter of 149 ± 12 nm and PDI of 0.06, as measured by DLS (Fig. 1a) and scanning electron microscopy revealed a monodisperse size distribution of spherical nanoparticles (Fig. 1b). The nanoparticles solutions proved to be very stable, with a zeta potential of – 29 ± 3 mV (Fig. 1c), measured by Zetasizer nano ZS.

**Figure 2:**
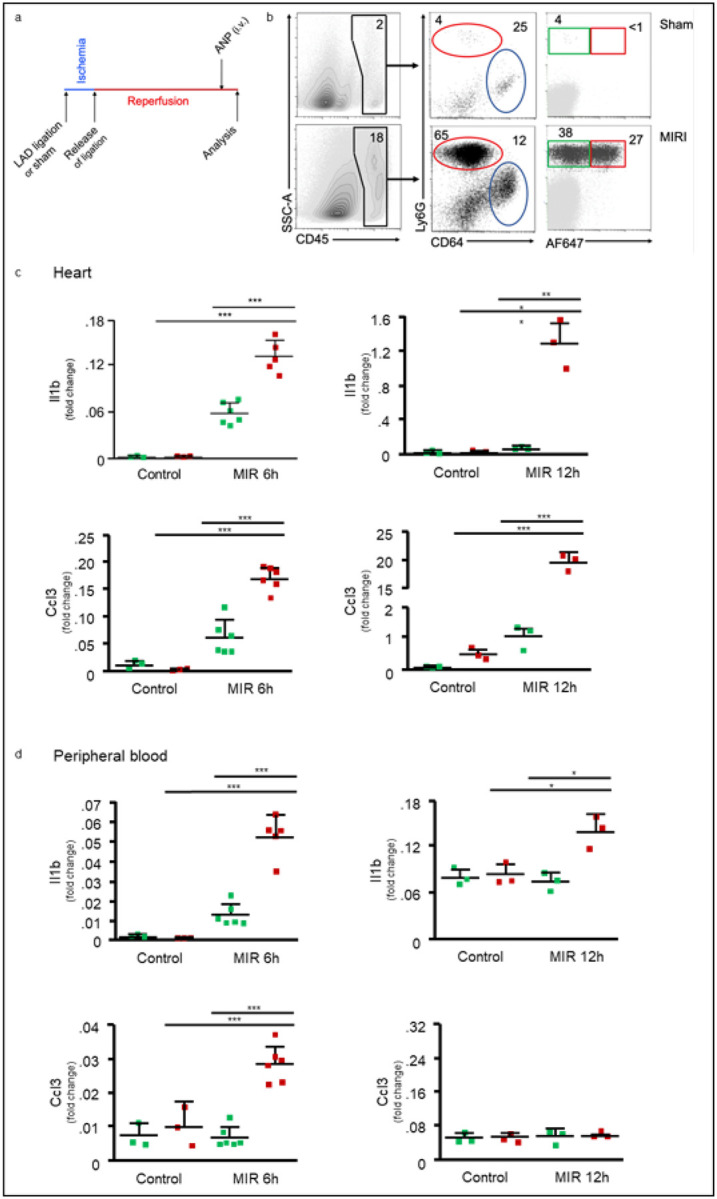
Myocardial IR injury in mice causes acute innate inflammation characterized by sequestration of distinct subsets of neutrophils. Flow cytometric analysis of heart cells from sham operated mice or after IR injury. **(a)** Scheme of IR injury induction and i.v. ANP-injection. Ligation was released 45’ post LAD ligation; cardiac inflammation was analyzed 6h or 12h after LAD ligation or sham operation. ANP were inject 30’ prior to euthanasia. **(b)** IR injury caused massive inflammation (CD45^pos^ cells increased from 2% in sham control hearts to 18%). ANP endocytosis is mostly restricted for Ly6G-expressing neutrophils; neutrophils with low or no endocytosis of albumin nanoparticles (ANP^low^, green), or neutrophils that readily endocytosed albumin nanoparticles (ANP^high^, red). Heart single cell suspensions were prepared, and cells were stained with specific antibodies to Ly6G (neutrophils), CD64 (monocytes and macrophages), CD45 (all leukocytes), and ANP labeled with AF647. Percentages of cell populations are indicated. **(c,d)** Il-1b or Ccl3 mRNA expression 6h or 12h after sham operation or IR injury induction. RNA for qPCR was prepared from peripheral blood Ly6G-positive ANP^low^ or ANP^high^, neutrophils. Ly6G-expressing neutrophils were selected (via magnetic beads) from single cell suspensions of peripheral blood or heart and sorted by flow cytometry according to their endocytosis of ANP. Fold change, expression, using Il-1b- or Ccl3-specific primers, relative to Ppia- (6h) or 18S rRNA- (12h) genes, determined by comparative Ct method. Markers represent results from individual mice. ***p< 0.0001, **p< 0.001, *p< 0.01 (unpaired t-test). Representative data from 3 independent experiments are shown.

We tested these albumin nanoparticles (ANP) *in vivo* using a well-established mouse model of IR injury (38). Experimental IR (1h ischemia, followed by 11h reperfusion) elicited an acute inflammatory response (Fig. 2a), with massive cardiac inflammation (CD45^+^ cells increased from 2% in sham control hearts to 18%) (Fig. 2b). The inflammatory cells were mostly Ly6G^+^ neutrophils and CD64^+^ monocytes and macrophages (Fig. 2b). Intravenous injection of ANP after 30’ prior to euthanasia showed that ANP endocytosis was largely restricted to the Ly6G-expressing neutrophils (Fig. 2b). Furthermore, ANP injections showed two distinct subsets of heart inflammatory neutrophils: neutrophils with low or no endocytosis of ANP (ANP^low^) and neutrophils that readily endocytosed ANP (ANP^high^) (Fig. 2b).

We next examined whether inflammatory mediators implicated in the pathogenesis cardiac IR injury were differentially expressed in these two neutrophil subsets. We isolated RNA of heart inflammatory ANP^low^ and ANP^high^ neutrophils, and quantified Il-1β expression by qPCR. We found that ANP^high^ neutrophils expressed significantly more mRNA for Il-1β than ANP^low^ neutrophils (Fig. 2c). The chemokine Ccl3 (Mip1α) is associated with the inflammatory response accompanying IR injury (39) and was induced 6h and 12h after the induction of IR injury. Heart ANP^high^ neutrophils expressed much greater amounts of Ccl3 than heart ANP^low^ neutrophils (Fig. 2c). Differential endocytosis of ANP was also evident in peripheral blood neutrophils. Here too, we found that peripheral blood ANP^high^ neutrophils expressed significantly more Il-1β and Ccl3 mRNA than ANP^low^ neutrophils 6h after initiation of IR injury (Fig. 2c). At 12h after IR injury, Il-1β was differentially expressed between the two neutrophil-subsets, whereas induction of Ccl3 expression in neutrophils was no longer apparent at 12h after IR injury (Fig. 2c), suggesting that targeting toxic neutrophils would be most effective within the initial 6h period of reperfusion. We identified two subsets of neutrophils by their selective endocytosis of ANP. In the peripheral blood and in inflamed myocardium, ANP^high^ neutrophils expressed inordinate amounts of inflammatory mediators implicated in promoting neutrophil adhesion and transmigration and in the pathogenesis of lethal cardiac reperfusion injury (40).

### Selective endocytosis of ANP enables treatment of cardiac IR

We next tested whether selective endocytosis of ANP by neutrophils could be utilized for the reduction of cardiac inflammation post IR. IR injury leads to the release of DAMP-receptors and the activation of downstream signaling molecules such as the spleen tyrosine kinase (Syk) (41). Syk has diverse functions (42) and is essential in CD18 integrin signaling in neutrophils (43, 44), and for the induction of cytokine and chemokine expression (45). Albumin nanoparticles can function as efficient carriers of therapeutics (46–48). We utilized a specific Syk inhibitor, the hydrophobic agent piceatannol, for delivery via ANP to neutrophils. To functionalize ANP with piceatannol, we incubated purified HSA (20 mg/mL) with piceatannol (5mg) prior to the desolvation reaction (Extended data Fig. 1). Drug encapsulation efficiency (measured by LC-MS/MS) was 6.1 ± 0.35% for reactions incubated with 5 mg of piceatannol, which effectively delivered 30.3 ± 1.7 μg/mL piceatannol at the piceatannol-ANP (PANP) formulation concentration of 2 mg/mL. We used these PANP formulations (concentrated solutions in deionized water of 15 mg piceatannol/g albumin, stable at 4°C for more than a month) for all therapeutic experiments.

**Figure 3:**
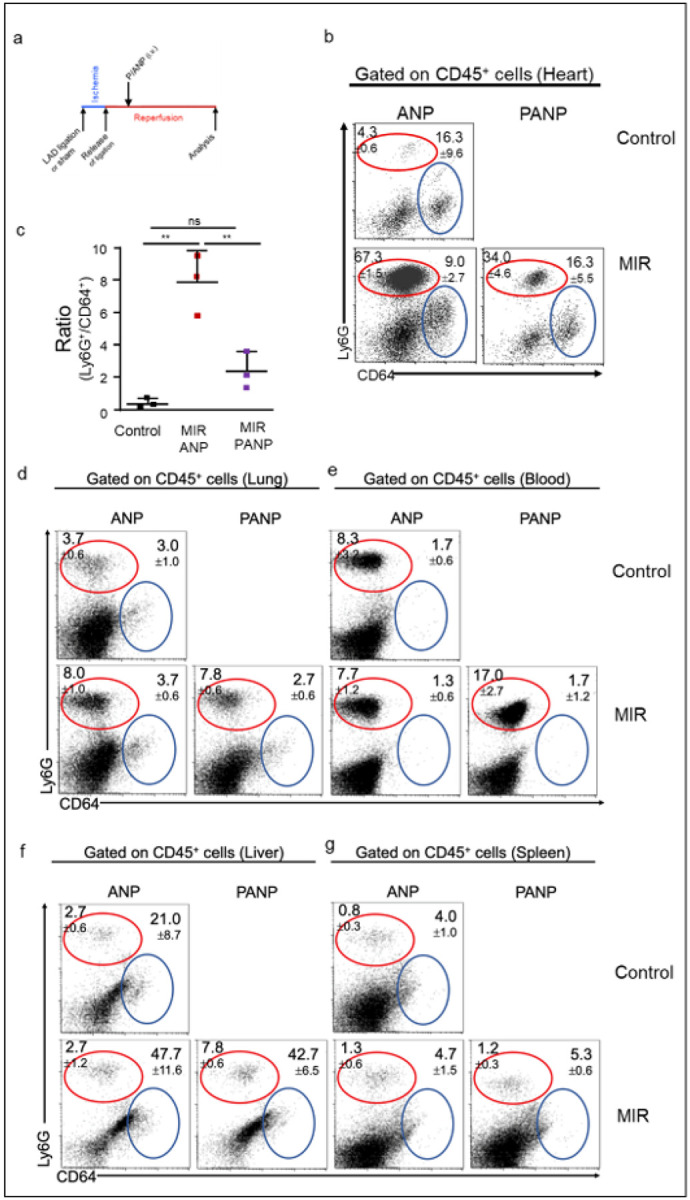
PANP treatment after IR reduces myocardial inflammation. Flow cytometric analysis of heart single cell suspensions from mice sham operated or after IR injury injected *i.v*. with ANP (vehicle control) or PANP (piceatannol). **(a)** Scheme of PANP treatment. Ligation was released 45’ post LAD ligation ANP or PANP were injected *i.v*. 1h after reperfusion. Innate cardiac inflammation was analyzed 24h after LAD ligation or sham operation. Massive inflammation in hearts of vehicle ANP-treated mice **(b)**. Markedly reduced myocardial inflammation of Ly6G^+^ neutrophils and CD64^+^ monocytes and macrophages in hearts of PANP-treated mice **(b)**. PANP-treatment significantly reduced ratio of Ly6G^+^ neutrophils to CD64^+^ monocytes and macrophages to level of sham-operated controls **(c)**. Sterile inflammation caused by IR injury was also seen in lungs **(d)** and PANP treatment correlated with increased percentage of Ly6G^+^ neutrophils in peripheral blood **(e)**. Percentage of Ly6G^+^ neutrophils and CD64^+^ monocytes and macrophages in liver **(f)** and **(g)** spleen was similar in all three treatment cohorts. Mice were euthanized for analysis 24h after initiation of IR injury or sham operation. Percentages of cell populations are indicated. Representative data from 3 independent experiments are shown.

We induced ischemia via LAD-ligation, released the ligation after 1h, and administered PANP or vehicle loaded ANP *i.v*. another hour after the release of the LAD-ligation; we euthanized the mice 24h after initiation of IR injury for analysis (scheme in Fig. 3a). We found that PANP treatment drastically reduced myocardial inflammation of Ly6G^+^ neutrophils and CD64^+^ monocytes and macrophages (Fig. 3b). The ratios of myocardial Ly6G^+^ neutrophils to CD64^+^ monocytes and macrophages were significantly reduced in hearts of PANP-treated mice to the same level as seen in untreated sham controls (Fig. 3c). Reduced cardiac inflammation after PANP-treatment had no effect on the percentage of Ly6G^+^ neutrophils in the lung (Fig. 3d), correlated with an increased percentage of Ly6G^+^ neutrophils in the peripheral blood (Fig. 3e) and the liver (Fig. 3f). The spleens from sham-operated or after IR injury treated with ANP or PANP, showed similar percentages of Ly6G^+^ neutrophils and CD64^+^ monocytes and macrophages (Fig. 3g). These data demonstrated that piceatannol could be effectively delivered via PANP and that this selective delivery targeting a distinct subset of neutrophils effectively reduced overall myocardial inflammation after IR without causing inflammation elsewhere.

**Figure 4:**
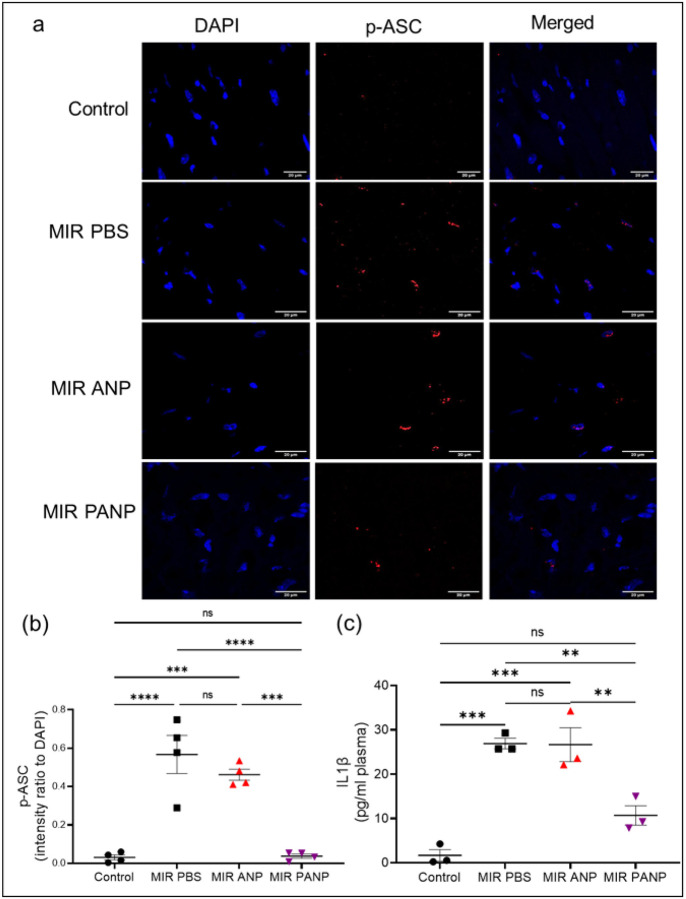
PANP treatment after IR curbs NLP3 inflammasome activation and IL-1b maturation. **(a)** Histological sections of hearts from control or MIR-subjected mice injected with PBS, ANP or PANP. The sections were stained with an antibody to p-ASC (red) and DAPI (blue) and imaged by confocal microscopy. Representative confocal microscopy images. **(b)** Quantification of p-ASC intensity with respect to DAPI per field of view as calculated by ImageJ software. **(c)** Quantification of IL-1β in plasma collected from control mice and mice after MIR injected with PBS, ANP and PANP as measured by ELISA assay. *p< 0.05; **p< 0.005; ***p< 0.0005 (One-way Anova followed by Tukey’s multiple comparison test in GraphPad Prism). Markers represent data from individual mice.

Cardiac-derived DAMPs prime and activate of the NOD-like receptor protein 3 (NLRP3) inflammasome (49). NLRP3 inflammasome processes the product of the Il-1β gene and is essential for its release from the cells (50). NLRP3 inflammasome function is dependent on recruitment of the adaptor molecule apoptosis-associated speck-like protein containing a caspase recruitment domain (ASC). ASC activation (ASC phosphorylation, p-ASC formation), is required for caspase-1-dependent processing of pro-IL-1β to mature IL-1β (51). After induction of IR injury, ASC phosphorylation (p-ASC formation), was apparent in inflammatory cells and absent in parenchymal cells (Fig. 4a,b). Moreover, plasma IL-1β concentration increased over controls in mice after IR-injury (Fig. 4c). Treatment of mice with vehicle ANP had no significant effect on inflammasome activation or secretion of mature IL-1β (Fig. 4a–c) but treatment with PANP significantly reduced p-ASC formation and, remarkably, reduced the concentrations of IL-1β in plasma to the levels in naive controls (Fig. 4a–c). These data indicate that the inhibition of Syk, specifically in the subset of ANP-endocytosing neutrophils is sufficient to reduce NLRP3 activity (p-ASC formation) and to reduce the release of mature IL-1β into circulation.

**Figure 5:**
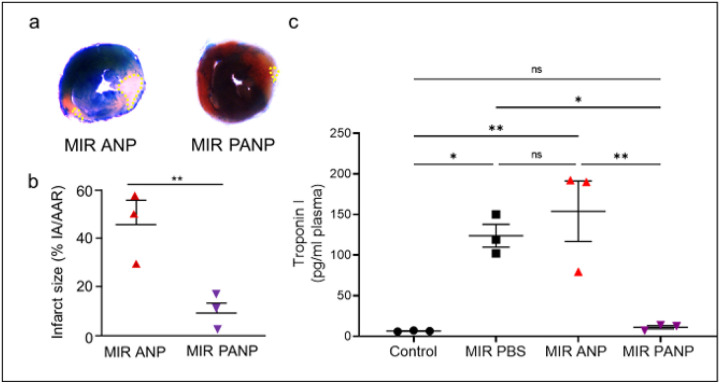
PANP treatment post IR reduces myocardial infarct size and cardiomyocyte death. **(a)** TTC staining of hearts from ANP control- or PANP-treated mice at 24h after initiation of IR injury. Dotted yellow lines highlight areas of dead myocardium. **(b)** Quantification of data shows that the infarcted area (IA) relative to area at risk (AAR) was significantly reduced by PANP treatment. Mice were treated with ANP or PANP, and at 24h after MI, hearts were harvested and the area of infarct - area at risk ratio (IA/AAR) was evaluated by dual staining technique. **p< 0.001 (Unpaired t-test). Representative data from 3 independent experiments are shown. **(c)** Troponin I plasma concentrations were significantly reduced by PANP treatment when compared to saline or ANP-vehicle treated mice and similar to concentrations found in naive control mice. *p< 0.05; **p< 0.005 (One-way Anova followed by Tukey’s multiple comparison test in GraphPad Prism). Markers represent data from individual mice.

We next investigated whether PANP treatment would also reduce infarct sizes. We induced IR injury and determined the infarcted area (IA) relative to the area at risk (AAR) in hearts excised immediately after euthanasia at 24h after initiation of the initiation of IR injury. IR resulted in massive myocardial necrosis in hearts of ANP-treated control mice (Fig. 5a). When compared to ANP-treated controls, PANP treatment preserved much of the LV myocardium (Fig. 5a) and markedly reduced infarct area: area at risk ratio (IA/AAR) compared to ANP-treated controls (45.6 ± 8.6 vs 9.2 ± 4%) (Fig. 5b). Infarct size in experimental models as in patients can also be assessed by the extent of the release of biomarkers, e.g., the cardiomyocyte-specific troponin I (52, 53). Consistent with our findings in infarct size, Troponin I concentrations were significantly reduced by PANP treatment when compared to saline or ANP-vehicle treated mice. Remarkably, plasma Troponin I concentrations of PANP-treated mice were similar the concentrations found in plasma of naive controls (Fig. 5c). We also evaluated cardiomyocyte death by *in situ* TUNEL staining (54). Consistent with our findings on infarct size and Troponin I concentrations, we observed a marked reduction in cardiomyocyte death by PANP treatment (Extended data Fig. 2).

### PANP treatment post IR preserves cardiac function

**Figure 6:**
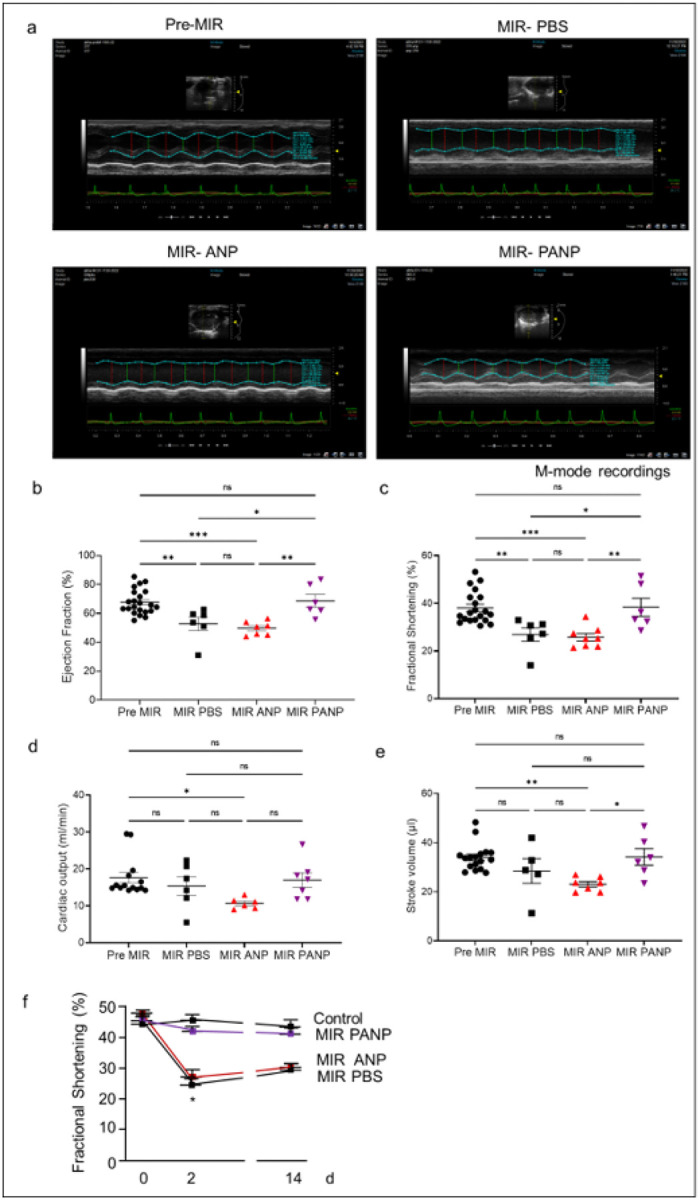
PANP treatment post IR preserves cardiac function. **(A)** Representative echocardiography imaging of hearts from controls and mice treated with ANP or PANP. We determined **(B)** ejection fraction, **(C)** fractional shortening, **(D)** cardiac output, **(E)** stroke volume in short axis echo imaging. *p< 0.05; **p< 0.005; ***p<0.0005 (One-way anova followed by Tukey’s multiple comparison test in GraphPad Prism). Marker represent data from individual mice. **(F)** LV fractional shortening was determined before (basal value, 0 time) and 2d and 14d after initiation of IR injury. Mice were treated with ANP or PANP 1h after the release of LAD ligation. Vehicle treated (ANP) mice suffered a marked decrease in shortening at 2d and 14d compared to PANP treated mice or sham controls. Sham (n=5), ANP (n=8) and PANP (n=5). Fractional shortening in ANP treated mice was significantly (*p<0.01 Prism 8) below sham and PANP treated animals at 2 and 14 days. Statistics were determined by 2-way Anova followed by Tukey’s multiple comparison test (GraphPad Prism). Representative data from 3 independent experiments are shown.

**Figure 7:**
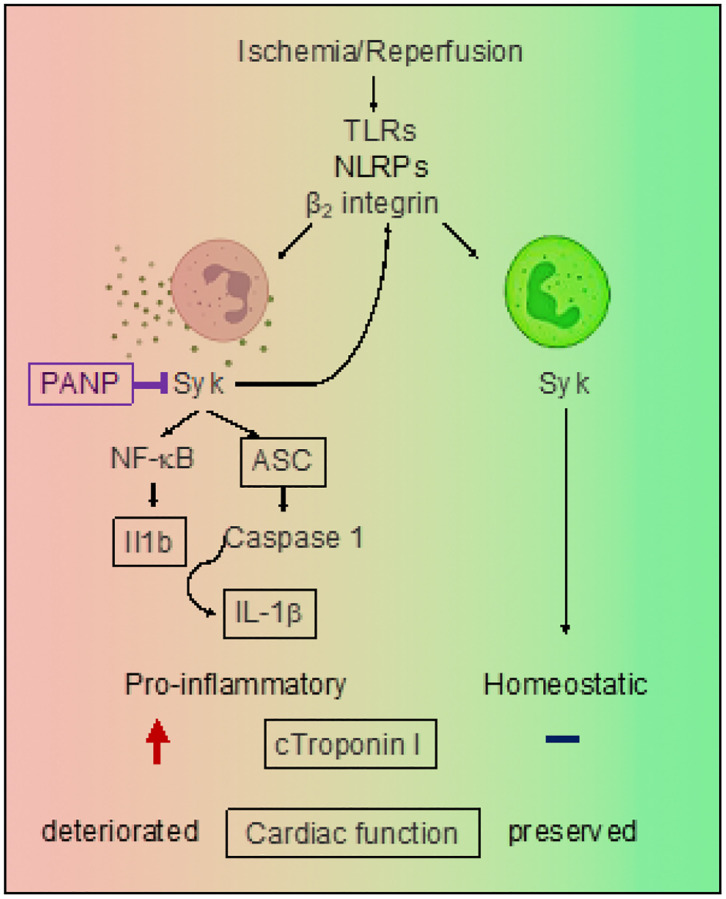
Graphical summary of findings. IR injury activates neutrophils, leading to neutropoiesis and functionally diverse inflammation of the myocardium. This study reveals that neutrophils with tissue-toxic potential are main contributors to IR. Therapeutic targeting of the inflammatory Syk pathway specifically in these cells mitigates the myocardial damage caused by IR. Notably, pharmacological Syk inhibition in these cells reduces cardiomyocyte death and preserves cardiac function post IR-injury.

To ascertain the effects of preventing IR-injury on left ventricular (LV) function, we determined indices of cardiac function, fractional shortening (FS), ejection fraction (EF), stroke volume (SV), and cardiac output (CO). We compared baseline function to function 24h post initiation of IR injury. We found that PANP treatment prevented deterioration of FS, EF, SV and CO (Fig. 6a–e) when compared to baseline cardiac function. We next determined LV fractional shortening before (basal value, 0 time) and 2d and 14d after initiation of IR injury. Injection of vehicle ANP did not prevent the worsening of LV fractional shortening echocardiographically measured 2d after induction of IR injury (Fig. 6f) whereas treating mice with PANP preserved fractional shortening (with values similar to sham-operated controls) (Fig. 6f). The beneficial effects of PANP treatment persisted 2 weeks after surgery (Fig. 6f), suggesting that PANP treatment shown to be beneficial in the early stages of recovery did not induce adverse cardiac remodeling and functional deterioration later. We additionally assessed cardiac myofiber disorganization, indicative of incipient adverse remodeling. Cardiac myofiber disorganization was markedly ameliorated by PANP treatment post IR (Extended data Fig. 3). These results suggest that mitigating IR injury by specific targeting of a neutrophil subset after reperfusion is beneficial in experimental MI (Fig. 7).

## Discussion

Efforts to target neutrophils therapeutically in patients with MI have shown unsatisfactory results in clinical trials (20–22). Since then, a deeper understanding of neutrophil heterogeneity (30, 33, 55, 56) have revived an interest in targeting specific neutrophil subsets to improve therapy of patients with acute coronary syndrome (29). We have shown here that a subset of inflammatory injury-inducing neutrophils can be specifically targeted to dampen the function of these cells and prevent myocardial injury and improve cardiac function post IR. These new results are in contrast to results of earlier experimental studies and clinical trials concentrating on interfering with all neutrophils, irrespective of their distinct functions, and which resulted in harmful outcomes (20–22). Our new results demonstrate the effectiveness of neutrophil subset-targeted therapy. The advantage of the present approach is the targeting of a specific neutrophil population. Non-targeted approaches to inhibit neutrophil function or recruitment have the potential to interfere with the beneficial effects of distinct neutrophil subsets and other inflammatory cells (57–61).

In the healthy individual neutrophils are produced in vast numbers as they are essential defenders protecting the host against a polymicrobial environment. It has become evident that neutrophils function to preserve tissue homeostasis (30), they are also toxic effectors and can cause irreparable harm to host tissues (62). Whether these seemingly paradoxical functions are due the existence of neutrophils as *bona fide* subsets with distinct functions or divergent responses to distinct micro-environments is still unclear (63, 64). In cardiac IR injury, neutrophils are involved both in causing cardiomyocyte death and in preserving heart tissue homeostasis (7, 16, 65). Indiscriminate inhibition of all neutrophils will affect with both these functions. To obviate this concern, we took advantage of neutrophil heterogeneity to change the toxic subset of neutrophils while preserving the neutrophils essential for tissue-homeostasis. Earlier reports demonstarted a phenotypic and functional profile of tissue-toxic ANP^high^ neutrophils that exist side by side with antimicrobial ANP^low^ neutrophils (33). Neutrophil recruitment in acute inflammation is mediated largely by the β_2_-integrin family of receptors, with Mac-1 (αMβ_2_, CD11b/CD18) and LFA-1 (αLβ_2_, CD11a/CD18) being the chief among them (44). The Syk tyrosine kinase is essential in CD18 integrin signaling in neutrophils (42, 43). Syk functions in both ANP^high^ and ANP^low^ neutrophils. IR injury caused acute inflammation with a substantial portion of ANP-endocytosing (ANP^high^) neutrophils. Inhibition of “outside-in” signals generated by engagement of β_2_-integrin in the subset of ANP^high^ with piceatannol reduced overall myocardial inflammation and rebalanced the ratio of neutrophils to monocytes and macrophages in the myocardium of treated mice. Similarly, piceatannol delivered to infiltrating neutrophils in a mouse model of acute ischemic stroke was associated with decreased stroke size (66, 67). The clinical use of Syk-inhibitors in the course of sterile cardiac inflammation is thus potentially therapeutic when it can be restricted to the tissue toxic neutrophil subsets.

Experimentally, heterogeneous chemokine receptor expression was used to delineate a subset of aged neutrophils (68), and a subset of aged neutrophils in the circulation was detrimental to myocardial tissues after vascular ischemia and reperfusion (69). Cardiac damage, as measured by infarct size, varied diurnally (69), a finding that was attributed to circadian changes in the armamentarium of neutrophils (70). Since the mice in the present study were of the same age and genotype we cannot ascribe differences in neutrophil function to these factors. Protection from myocardial injury could also be achieved by pharmacological targeting of a member of the Src family of protein tyrosine kinases, *Fgr*, or by using of *Fgr* gene-deficient mice (71). In the latter study it was claimed that cardiac protection depended on targeting of *Fgr* specifically in neutrophils (71). It should be noted that *Fgr* is significantly higher expressed in activated ANP^high^ than in ANP^low^ neutrophils (33), thus we cannot rule out the role of *Fgr* in the observed protection.

Clinically, cardiomyocyte-protective strategies aimed at preventing cardiomyocyte death have produced mixed results (72, 73). A large randomized clinical study completed a decade ago (the CIRCUS trial) could not confirm earlier findings (74) on the cardioprotective effects of cyclosporine administered to patients with acute myocardial infarction before percutaneous coronary intervention (75). Activation of sphingosine receptors induced cardioprotection both *in vitro* and *in vivo* (76), but clinical efficacy of the sphingosine-1-phosphate mimetic drug fingolimod (FTY720) was not demonstrated (76). A clinical study demonstrated that brief cycles of non-lethal ischemia and reperfusion applied to the upper arm down-regulated the expression of kinin B1 and B2 receptors in neutrophils of patients undergoing cardiac surgery (3, 77), possibly reducing cardiac neutrophilic inflammation. Preconditioning, postconditioning, and remote conditioning of the myocardium enhanced the ability of the heart to withstand IR insults (78, 79), but large multicentered randomized studies confirming or refuting these findings on clinical outcomes are still missing (80, 81). Drugs that enhance nitric oxide (NO) release (e.g., statins, calcium antagonists, ACE-inhibitors, dexamethasone), NO, or NO donors had to be administered prior to ischemia to protect the myocardium against IR injury (82, 83), making their clinical use problematic.

The innovation of the nanotherapeutic approach as described is that it preserves the beneficial effects of neutrophils and other innate inflammatory cells in protecting myocardial integrity in the immediate aftermath of MI (Fig. 7). Successful therapies targeting post-ischemia-reperfusion cardiovascular inflammation will require precision and specificity. Our results warrant clinical studies that may identify human neutrophil subsets of similar phenotype and function to those we identified in mice. This study reveals that neutrophils with tissue-toxic potential contribute significantly to cardiac IR injury because targeting the inflammatory Syk pathway specifically in these cells mitigates the myocardial damage caused by IR.

## Methods

### Preparation of albumin nanoparticles (ANP and PANP).

Human serum albumin (HSA, MW 66,500 Da) was purchased from Akron Biotech and purified by acetone and 0.2μm filter. Glutaraldehyde (25% in water) was bought from Sigma Aldrich. HSA concentration was measured using the Coomassie (Bradford) Protein Assay Kit (Fisher Scientific). ANP were prepared following a desolvation technique, in a modification of an earlier technique (32). Purified HSA was diluted to 20mg/mL with endotoxin free water. The HSA solution (1mL) was transformed into nanoparticles with addition of pure ethanol (3.5mL) over ten minutes while stirring at room temperature and stabilized by the addition of 38μL glutaraldehyde left to stir for a minimum of 4 hours. ANP were collected by centrifugation (15,000 g, 20min, 4°C.) and washed three times by resuspension in endotoxin free water (1mL). After the third wash, the pellet was resuspended in high concentration (~ 20g/mL) and stored at 4°C prior to formulation for experiments. For PANPs piceatannol (5mg) was dissolved in DMSO (50μL) by strong agitation, which was then added to the HSA solution (20mg HSA, 1mL endotoxin free water). The mixture was left stirring to incubate for a minimum of 1 hour, allowing the piceatannol to interact with the solubilized HSA. The mixture remained covered in foil to prevent UV degradation of piceatannol. After 1h, the synthesis continued following the ANP with the addition of ethanol and glutaraldehyde. Loading efficiency was measured via extraction followed by LCMS (Alliance 2795 HPLC, Quattro micro API triple quad (QQQ) mass spectrometer, Waters, Milford, MA, USA.). Extraction was carried with acetonitrile using an internal standard working solution (custom synthesized trans-Piceatannol-d3, 10mg/mL, 20μL). Piceatannol (98%) was purchased from MuseChem and trans-Piceatannol-d3 was custom synthesized by Toronto Research Chemicals Inc. The endotoxin content of prepared nanoparticles was measured using a Genscript ToxinSensor Chromogenic LAL Endotoxin Assay Kit. Endotoxin content was found to be 0.109 Eu/mL for infused nanoparticle formulations at a nanoparticle concentration of 2mg/mL. Alexa-647 (NHS ester), acetone (ACS Grade), ethanol (200 proof), water for injection (WFI), endotoxin free water (HyClone), and phosphate buffered saline (PBS, 1x, without magnesium or calcium) was purchased from Fisher Scientific. Alexa-647 (25μg) was dissolved in DMSO (10μL). Then, the Alexa-647 solution was added to the HSA-piceatannol solution and incubated for one hour. The synthesis then proceeded as before with the addition of ethanol. Any unloaded dye was washed out of the product during the three consecutive washes at the end of the procedure. PANP were characterized by determining size, polydispersity index (PDI), and surface charge as zeta potential. Size and PDI were measured via dynamic light scattering using a Zetasizer (ZS, Malvern Industries, Worcestershire, UK.). First, the sample was diluted with 0.2μm filtered water (1mL). Then, a disposable cuvette was filled with 0.2μm filtered water (1mL). Next, seven drops of sample were added, and the cuvette was shaken lightly to mix. Finally, the cuvette was placed in the Zetasizer and measured. Zeta potential was measured by laser Doppler micro electrophoresis using a Zetasizer. The previous sample was diluted with 0.2μm filtered water (1mL). The sample was then added to a disposable folded capillary cell and measured. Size was verified via nanoparticle tracking analysis using a Nanosight (NS3000, Malvern Industries, Worchestershire, UK.) The sample was first diluted 100,000x with 0.2μm filtered water. Then the sample was injected into the Nanosight via syringe pump and read at 500nm to obtain a video and analyzed to give particle size distribution.

### Mice.

We used C57BL/6 male mice, body weight 25 to 28g. Procedures were approved by the Institutional Animal Care and Use Committee of University of Illinois, Chicago. Anesthesia was introduced with 1.5–3% isoflurane inhalation in a closed glass chamber and Etomidate (10 mg/kg body weight IP). Mice were orally intubated with a 18G angiocath sleeve and artificially ventilated with a rodent respirator (tidal volume 0.2–0.3ml (based on body weight), rate 135 strokes/min). Surgical anesthesia was maintained using 1% isoflurane delivered through a vaporizer with air connected in series to rodent ventilator. A dose of buprenorphine sustained release (1.0 mg/kg, *s.c*.) was administered pre-operatively for long term analgesia.

### Flow Cytometry.

Single cell suspensions were prepared as described (84, 85). Cells were stained for 30 min on ice. Dead cells were excluded by F-SC, S-SC. Neutrophils were gated by Ly6G^hi^ S-SC^hi^. Antibodies as listed ere used. Isotype-matched Abs to irrelevant epitopes were used as negative controls. Isolated Ly6G^+^ peripheral blood or heart cells were sorted into nanoparticle-endocytosing or -non-endocytosing cells according to their nanoparticle-specific fluorescence using a MoFlo Astrios cell sorter. Reagents used for flow cytometry:

**Table:** 

Ab to	Clone	Source
CD45	30-F11	Biolegend
Ly6G	1A8	Biolegend
CD31	390	Biolegend
CD11b	M1/70	Invitrogen
CD11c	N418	Biolegend
CD16	2.4G2	Biolegend
LY6C	HK1.4	Biolegend
CD64	X54–5/7.1	Biolegend
F4/80	BM8	Invitrogen
MHC11	M5/114.15.2	Biolegend
MERTK	2B10C42	Biolegend
CD206	C068C2	Biolegend
**Viability dye** Zombie Aqua		Biolegend

### Myocardial infarction and ischemia reperfusion injury (IR injury) and nanoparticle administration.

Fully anesthetized mice were intubated and kept at a controlled temperature of 37°C throughout the experiment. Mice were subjected to 1h occlusion of the left anterior descending (LAD) coronary artery followed by reperfusion. Thoracotomy was performed by 1 cm incision 1 mm to the left from a midline between the 2nd and 4th rib in layers. The LAD was ligated with 8 − 0 prolene suture 2 mm below the ostium. The effectiveness of the occlusion was verified by whitening of the ventricle distal of the ligation. The chest cavity was closed with 6 − 0 suture for 45 minutes and then reopened and the arterial ligation was removed. The chest cavity was closed with the 6 − 0 silk sutures. The skin was closed by 6 − 0 Polypropylene monofilament suture. The thorax was drained with PE-10 cannula inserted into incision upon closure. The tube was then removed, and suture completed. Post-operative analgesia was given peri-operatively with one dose of 1.0 mg/kg of buprenorphine sustained release SC. Mice were treated with PANP and ANP vehicle. HSA or BSA nanoparticle preparations were used interchangeably because we observed was no difference in efficacy or safety between preparations. Administration of drug was via tail vein or retroorbital injection at 1 after the removal of the ligation from the LAD with ANP or PANP (20 mg/kg PANP, delivering 0.03 mg/kg piceatannol). Wellbeing of mice was monitored over the course of the experiments.

### Evaluation of myocardial function and pathology.

For quantification of infarct size, we re-anaesthetized and re-intubated the mice, and re-occluded the LAD coronary by ligating the suture in the same position as the original infarction. Then, animals were euthanized and 1 ml of 1% Evans Blue dye (Sigma) was infused IV to delineate the area at risk (AAR: myocardium lacking blood flow, that is, negative for blue dye staining). To delineate the infarcted (necrotic) myocardium, slices were incubated in triphenyltetrazolium chloride (TTC, Sigma) at 37°C for 15 min. The slices were then re-photographed, weighed, and regions negative for Evans Blue staining (AAR) and for TTC (infarcted myocardium) were quantified. Percentage values for AAR and infarcted myocardium were corrected to mg independently for each slice. Absolute AAR and infarct size were determined as the mg:mg ratio of AAR:LV and infarcted myocardium:AAR, respectively) (86). Outcome assessment was performed blind to treatment. Troponin I (cTnI) and IL-1β concentrations were measured using commercial ELISA assay kits according to the manufacturers’ instructions.

### Echocardiography.

Animals are placed on a heated platform and kept at a controlled temperature of 37°C throughout monitoring (VisualSonics, Inc.) where ECG (lead II), heart rate, respiratory waveform and respiratory rate are monitored and displayed on the ultrasound’s CPU display. Nair depilatory cream was used to remove the hair in the vicinity of the chest. Prewarmed (37° C) acoustic coupling gel was then applied to the chest and the ultrasound probe used to obtain recordings. We measured cardiac function before 24h post occlusion. Mice were anesthetized by 3.5% isoflurane and placed a heated platform (VisualSonics, Inc.). Three views were recorded with the sample volume in the proper modes (B-mode, M-mode, pulsed Doppler or tissue Doppler): 1. The parasternal long axis [B-mode and/or M-mode]; 2. The parasternal short axis [M-mode was taken at the papillary level followed by tissue Doppler of the myocardium]; and 3. Apical view [pulsed Doppler of the mitral flow]. Pressure-Volume Loop analyses of Left Ventricle: under the same anesthetic regiment as for the MI procedure, a 1.4 French pressure-conductance catheter (SPR-839, Millar Instruments, Houston TX) was inserted into the right carotid artery to measure baseline arterial pressure, then advanced retrograde into the LV to record baseline hemodynamics in the closed chest configuration with the ARIA Pressure Volume Conductance System (Millar Instruments, Houston, TX). All data were analyzed with the PVAN 3.4 software package from Millar Instruments (Houston, TX).

### Histological analysis.

For histological analysis of Hearts from control mice and MIRI mice treated with PBS, ANP and PANP were fixed using 10% buffered formaldehyde and processed for paraffin section. The histological sectioning and staining for Hematoxylin & Eosin, Trichome staining and Tunel staining were done with the help of Research Histology Core (RHC), University of Illinois, Chicago. The quantification was done using ImageJ software. Further, the Aperio Image Scope system (Leica) was used to digitize histology slides and quantification.

### Statistical analysis.

Data were analyzed for statistical probabilities of differences using Graph Pad Prism 8 software. Continuous variables were expressed as mean ± standard deviation (SD) after normal distribution approximation was confirmed, and categorical variables as count and percentage. Continuous variables were compared among study groups with student’s t-tests followed by Scheffe’s adjustment for multiple comparisons. ANOVA test was carried out for an omnibus comparison among the four groups followed by Tukey’s multiple comparison test in GraphPad Prism. For the comparison of categorical variables, Fisher’s exact test was used. A p value of < 0.05 was regarded as statistically significant.

## Supplementary Material

Supplementary Files

This is a list of supplementary files associated with this preprint. Click to download.

- Extendeddatafigures.docx
